# A randomized, parallel study of the safety and efficacy of 45 mg primaquine versus 75 mg bulaquine as gametocytocidal agents in adults with blood schizonticide-responsive uncomplicated falciparum malaria [ISCRTN50134587]

**DOI:** 10.1186/1471-2334-6-16

**Published:** 2006-02-01

**Authors:** NJ Gogtay, KD Kamtekar, SS Dalvi, SS Mehta, AR Chogle, U Aigal, NA Kshirsagar

**Affiliations:** 1 Department of Clinical Pharmacology, Seth G.S. Medical College and K.E.M hospital. Parel, Mumbai 400 012, India; 2 Department of Medicine, Seth G.S Medical College & K.E.M Hospital, Parel, Mumbai 400012, India; 3 Kasturba Hospital for Infectious diseases, Sane Guruji Marg, Mumbai 400011, India

## Abstract

**Background:**

The WHO recommends that adults with uncomplicated P. falciparum successfully treated with a blood schizonticide receive a single dose of primaquine (PQ) 45 mg as a gametocytocidal agent. An earlier pilot study suggested that 75 mg of bulaquine (BQ), of which PQ is a major metabolite, may be a useful alternate to PQ.

**Methods:**

In a randomized, partial blind study, 90 hospitalized adults with Plasmodium falciparum malaria that was blood schizonticide-responsive and a gametocytemia of > 55/μl within 3 days of diagnosis were randomized to receive single doses of either PQ 45 mg or BQ 75 mg on day 4. We assessed gametocytemia on days 8, 15, 22 and 29 and gametocyte viability as determined by exflagellation (2° end point) on day 8.

**Results:**

On day 8, 20/31 (65%) primaquine recipients versus 19/59 (32%) bulaquine recipients showed persistence of gametocytes (P = 0.002). At day 15 and beyond, all patients were gametocyte free. On day 8, 16/31 PQ and 7/59 BQ volunteers showed gametocyte viability (p = 0.000065).

**Conclusion:**

BQ is a safe, useful alternate to PQ as a *Plasmodium falciparum *gametocytocidal agent and may clear gametocytemia faster than PQ.

## Introduction

Malaria remains the most important parasitic infection with 300–500 million people affected yearly and 1.5–2.7 million deaths each year. World over, malaria control has focused on pharmacological intervention, vector control, curtailing irrational and indiscriminate use of antimalarials, and the development of vaccines. Of these strategies, pharmacological intervention remains the most effective way to combat malaria [1]

8 aminoquinolines like primaquine are unique antimalarials in that they exhibit activity against multiple life cycle stages of *Plasmodia *that infect humans [2]. Primaquine 45 mg as a single dose is recommended by the World Health Organization (WHO) and the National Antimalarial Programme (NAMP) of India for its gametocytocidal activity in *P. falciparum*. [2] In 1998, Gogtay *et al *found that the efficacy of PQ 45 mg as a falciparum gametocytocidal agent in patients sensitive to chloroquine in India to be approximately 77% at Day 29 follow up. [3] During the past several years, attempts have been made to produce primaquine analogs with improved anti-malarial activity and lower toxicity. Bulaquine, formerly called CDRI 80/53, is metabolized to PQ and differs from PQ only by the 2,4 dihydrofuran group present in the basic side chain anchored onto the quinoline nucleus in the 8 position [4-6]. Bulaquine is currently licensed only for use in India for the radical cure of vivax malaria dosed at 25 mg/day for 5 days, but not as a gametocytocidal agent IN Plasmodium falciparum. The results of a pilot study by our group assessing the P. falciparum gametocytocidal effect of a single dose of bulaquine 75 mg in India suggested it may be more effective than PQ 45 mg. [7] Here, in a larger population of adults with P falciparum malaria successfully treated with blood schizonticides, we compared the gametocytodical activity of BQ and PQ.

## Volunteers and methods

### Protocol

The protocol was approved by the institutional ethics committee and the Drugs Controller General of India. The study was conducted between January 2002 and April 2004.

### Enrollment and procedures

Patients who were at least 16 years old with uncomplicated *Plasmodium falciparum *infection, provided written informed consent, and had a gametocyte count > 55/μl within 72 hours of diagnosis, regardless of asexual parasite counts, were eligible for enrollment. The minimal gametocyte count was chosen based on infectivity to mosquitoes [8]. Patients who were pregnant or lactating, had received antimalarial treatment in the previous 2 weeks, had co-infection with *Plasmodium vivax*, claimed an allergy to primaquine or bulaquine, or were G6PD deficient were excluded. On admission, patients were initially assessed by thick and thin blood films stained using the Jaswant Singh and Bhattacharji (JSB) field stain [9]. Subsequently, Giemsa stained blood smears were used to determine the number of asexual and sexual parasites/μl, assuming a white blood cell count of 8000/μl.

Enrolled patients were admitted to hospital on Day 1 and treated under observation with quinine orally 10 mg/kg/day thrice daily for a total of 7 days and doxycycline 100 mg once daily for 7 days. At day 4, consecutive patients were randomly allocated in a 1:2 fashion to receive an observed single dose of either PQ 45 mg or bulaquine 75 mg, based on a computer generated randomization code. Unequal allocation was used because of earlier studies suggesting the superiority of bulaquine. The test articles were administered on day 4 because the incidence of nausea and vomiting is higher in the first few days of schizonticidal therapy and this was given regardless of parasite clearance. on day 8, all patients were assessed for gametocytemia, discharged, and asked to follow up on days 15, 22, and 29 for further safety and parasitologic checks.

Giemsa stained malaria blood smears during hospitalization were done twice a day for the first 72 hours and once a day thereafter until discharge and on the follow-up days. The slide readers were blinded to the treatment.

### Outcomes

Efficacy was assessed by gametocytemia (primary end point) and gametocyte viability (secondary end point) on admission and all follow up days. The latter was assessed by the modified Shute's technique [10]. This technique depicts exflagellating microgametes in blood films that have been kept moist at 21–25°C for 1 hour with complete RPMI medium and AB positive serum and then Giemsa stained. One or more exflagellating microgametes was considered a positive test. These end points were identical to the previous study. [7]

Safety was monitored by routine clinical hematological and biochemical laboratories and an electrocardiogram on days 1 and 8. Adverse event recording was focused only on nausea, vomiting, and epigastric distress and were recorded only if not reported on Day 1 or if a symptom worsened after Day 1.

### Sample size and statistical analysis

The estimated sample size was calculated using Casagrande's method based on a previous study by Gogtay et al comparing the two drugs [7]. Assuming a 30% difference in efficacy on Day 8, at 5% significance and 90% power, a sample size of 28 patients and 56 patients are required in the primaquine and bulaquine group, respectively, to demonstrate the superiority of bulaquine. P values ≤ 0.5 were considered significant.

## Results

A total of 93 male patients were enrolled. Women with malaria are not inclined to get admitted, especially because of hardships related to hospitalization and supervised drug administration. The age of the patients ranged from 16–72 years: 31.47 ± 11.62 years). There were three drop outs, two in the Bulaquine arm and one in primaquine arm. These patients did not return for any follow up visit after discharge from the hospital and were omitted from analysis.

At admission, gametocytaemia between the 2 groups was similar. At day 8, 20/31 (65%) PQ recipients and 19/59 (32%) BQ recipients had gametocytes on blood smear (p = 0.002). At days 15, 22, and 29, all patients in both treatment groups were free of gametocytes.

At day 8, 16/31 (52%) PQ recipients and 7/59 (12%) BQ recipients had viable gametocytes on exflagellation testing (Table)(p = 0.000065). All patients with viable gametocytes had smear positive gametocytemia.

There were no important clinical hematology or biochemical laboratory values, and all electrocardiograms were within normal limits.

## Discussion

A single dose of 45 mg primaquine is given along with or after schizonticidal therapy in areas where malaria is endemic as a transmission blocking strategy and currently is the only option available for this indication. The present study carried out in 91 cases of uncomplicated *Plasmodium falciparum *malaria assessed the efficacy of bulaquine, the parent compound of primaquine. given as a single dose of 75 mg for its gametocytocidal effect. On day 8 of therapy, fewer patients with bulaquine had gametocytes as compared to those who received 45 mg primaquine. This suggests superior efficacy of bulaquine or its ability to clear gametocytes more rapidly, as all patients were free of gametocytes by day 15 and beyond.

This clearance of gametocytes completely by Day 15 in both groups in the present study contrasts with our previous study with 45 mg primaquine where persistent gametocytemia was seen in 23% patients responsive to chloroquine up to Day 29. [3] This may in part be due to use of different schizonticidal agents in the two studies, with chloroquine being used in the former and quinine in the latter, with quinine leading to greater clearance of asexual parasites.

Based on the results of the present study, bulaquine in a single dose of 75 mg may represent yet another treatment option for gametocytocidal effect in addition to primaquine and the current licensing of the drug in the country could be changed to include gametocytocidal effect apart from anti-relapse effect. Whether increasing the dose of primaquine from 45 mg to 60 mg will improve efficacy needs to be addressed in future studies.

In this study, the Shute technique, which measures the ability of the male gametocyte to exflagellate was used as a surrogate marker for assessing transmission blocking. Assessment of true gametocytocidal efficacy of any drug will depend upon demonstration of the ability to block transmission to mosquitoes. This in turn can be assessed by only checking for the presence of the oocyst and ookinete in the mosquito midgut [10], not done in the study.

In the first study where we first reported the declining efficacy of primaquine as a gametocytocidal agent, chloroquine versus coartemether was studied, since chloroquine then was (and still remains) the first line therapy for the country. [3] The results of the study showed a high degree of chloroquine resistance (> 50%), and we shifted to using quinine as first line for uncomplicated falciparum malaria in the hospital, since there are only isolated reports of quinine resistance in the country. Drugs that can block the spread of malarial parasites by killing or preventing the transmission or maturation of gametocytes, represent important tools in malaria control. Supputtamongkol et al compared the efficacies of mefloquine-artesunate and mefloquine-primaquine on the subsequent development of gametocytemias. [11] The latter combination was not found to be effective in either clearance of existing gametocytemias or the prevention of new gametocytemias. Primaquine has an extremely short half life, and as such may not be completely able to eliminate gametocytes and needs to be used with an effective schizonticidal agent. Persistence of gametocytemia is one of the predictors of treatment failure and use of an effective anti-malarial drug to eradicate asexual forms will still remain the most effective means to prevent gametocytemias.

**Table 1:** Gametocytaemia of patients at different days of follow-up in the two groups

Days of follow-up	Primaquine n = 31. Gametocyte density/μl(mean ± SD)	Bulaquine n = 59. Gametocyte density/μl(mean ± SD)
1	80-6040(1342 ± 182.80)	80-4160(1064.2 ± 182.80)
4	120-8000(1494 ± 385.04)	80-3260(1003.7 ± 118.35)
8	80-2120(543 ± 128.75)	32-1820(161.01 ± 32.76)
15,22,29	Nil	Nil

**Table 2:** Results of Efficacy.

Days of follow -up	No of patients positive for gametocytes		Patients sample positive for exflagellation	
	Pts treated with PQ N = 31	Pts treated with BQ N = 59	Pts treated with PQ N = 31	Pts treated with BQ N = 59

1	31/31 100%	59/59 100%	31/31 100%	59/59 100%
4	31/31 100%	59/59 100%	31/31 100%	59/59 100%
8	20/31 * 65%	19/59 * 32%	16/31 * 52%	7/59 * 12%
15	Nil	Nil	Nil	Nil
22	Nil	Nil	Nil	Nil
29	Nil	Nil	Nil	Nil

* statistically significant P < 0.05

Acknowledgements: This study was carried out as a project under the Center for Advanced Research in Clinical Pharmacology, and was funded by the Indian Council of Medical Research, New Delhi. We thank Nicholas Piramal India Ltd for the supply of bulaquine capsules.

## Pre-publication history

The pre-publication history for this paper can be accessed here:


